# GNE Myopathy: 25 Years After Gene Identification—Facts, Controversies, Enigmas, Prospects

**DOI:** 10.3390/jcm15176566

**Published:** 2026-08-25

**Authors:** Stella Mitrani-Rosenbaum, Zohar Argov

**Affiliations:** 1 Goldyne Savad Institute of Gene Therapy, Hadassah Medical Center, Faculty of Medicine, Hebrew University of Jerusalem, Jerusalem 91120, Israel; 2 Department of Neurology, Hadassah Medical Center, Faculty of Medicine, Hebrew University of Jerusalem, Jerusalem 91120, Israel

**Keywords:** sialic acid, GNE, animal models, metabolic therapy, myopathy therapy trials

## Abstract

Twenty-five years after our identification of the genetic defect in GNE Myopathy (GNEM), we review the current state-of-affairs in the research of this unique myopathy. In this narrative review, we describe the clinical aspects of this myopathy, the genetics of this muscle disorder, the biochemistry of the GNE enzyme, and the animal models that have been developed. We critically discuss the accumulating scientific and clinical data that show that hyposialylation cannot be the sole explanation for the disease pathomechanism. The negative or minimal effects of sialic acid supplementation in clinical therapy trials of GNEM call for a re-evaluation of future planned trials. We review the known facts and the current enigmas as well as research controversies in this field. We also discuss the prospects for further basic research, as a reliable animal model of GNEM is lacking, and future genetic therapy of this myopathy.

## 1. Introduction

In 1984, Argov and Yarom defined a muscle disorder entity in a Middle Eastern cluster of patients they termed Quadriceps Sparing Myopathy (QSM), and named later Hereditary Inclusion Body Myopathy (HIBM) [1]. In Japan a very similar clinical disorder was called Distal Myopathy with Rimmed Vacuoles (DMRV) [2]. In 2001, our group identified the causative genetic defect of this disorder in the Middle Eastern cluster. Mutations in the gene *GNE* were found to be the culprit defect in QSM/HIBM [3] and, soon after, the same gene with different pathogenic variants was found to be involved in DMRV [4]. Following our discovery numerous patients with different *GNE* mutations and a similar phenotype were diagnosed world-wide. A consortium of investigators (clinical and basic scientists) agreed to unify the different names of this condition in 2014, now called GNE Myopathy (GNEM) [5]. Although no systematic epidemiologic studies have been performed, a recent global estimation of 10 cases per million has been reported, using population genetic databases [6]. Twenty-five years after the identification of the causing gene, this unique condition is still not fully understood despite intensive research by several laboratories and clinical institutes [7,8,9]. In this review, based on historical comprehensive tracking as well as screening of the major literature databases up to 2026, we describe what is known (facts), what is still argued about (controversies), which facts cannot be well explained (enigmas), and what the GNEM field still needs to investigate (prospects). This narrative review reflects our perspective on the evolution of GNEM research over the past quarter of a century.

## 2. Clinical Aspects

Classical GNEM is an adult-onset progressive myopathy that starts as distal weakness in the legs and progresses to involve other muscles in the lower limbs, with marked sparing of the quadriceps and later spreading to the upper limbs and neck muscles. Muscle histology shows variable numbers of vacuolated fibers and associated tubofilamentous inclusions [1]. These histologic findings, which initially gave the disorder its various names, are not considered specific. Diagnostic biopsy is rarely performed today in suspected GNEM.

During the 25 years following the identification of the genetic basis of GNEM, the clinical spectrum of the disorder has widened [10]. There are a few clinical observations that occur infrequently in genetically proven GNEM (Table 1): onset in proximal leg muscles, onset in hand muscles, no sparing of the quadriceps, and mild facial weakness. Respiratory muscle weakness is rarely a clinical problem but was shown to occur in the advanced stages of the disease. Similarly sleep apnea was found to be more prevalent in GNEM patients than previously identified. We and others have seen very rarely unexplained cardiomyopathy in GNEM. The vast majority of GNEM patients have no clinical heart involvement, although, in some, reduced ejection fraction was observed in cardiac ECHO studies.

The main clinical enigma of GNEM is the quadriceps sparing, a feature that is observed not only clinically but also by muscle imaging. Quadriceps sparing exists even in advanced cases with full paralysis of limb muscles and it serves as a typical clinical diagnostic clue. Understanding this feature may point toward the existence of ‘protective mechanisms’ in this condition and help designing a therapeutic strategy. We believe that this feature is a result of post translational changes in the quadriceps muscle. Patients, however, are reluctant to have comparative biopsies of the quadriceps and typically affected muscle (tibialis anterior or biceps) for more advanced studies.

An additional unexplained observation is the fact that two elderly subjects homozygous for a typical *GNE* mutation (M743T) are completely asymptomatic, with no muscle weakness or any other muscle symptom, even in their 7th–8th decade of life [11], despite having close family members with the ‘classical’ form of GNEM. Unfortunately, we could not perform muscle biopsies on these subjects for investigational studies. The protective mechanism for this observation remains another ‘elusive’ enigma.

Another enigma related to GNEM is the rare *GNE*-associated macrothrombocytopenia with marked bleeding tendency in patients with two recessive *GNE* pathogenic variants [12]. This severe hematologic disorder is typically identified in early life, long before the age of onset of GNEM. Most GNEM patients have a normal (or slightly low) thrombocyte count [13]. It was suggested that hyposialylated thrombocytes have increased hepatic clearance [14] but this cannot explain the rarity of the macrothrombocytopenia in most GNEM patients, suggesting that an additional factor is responsible for the expression of the hematologic disorder in these individuals.

## 3. Genetics

**Figure 1:**
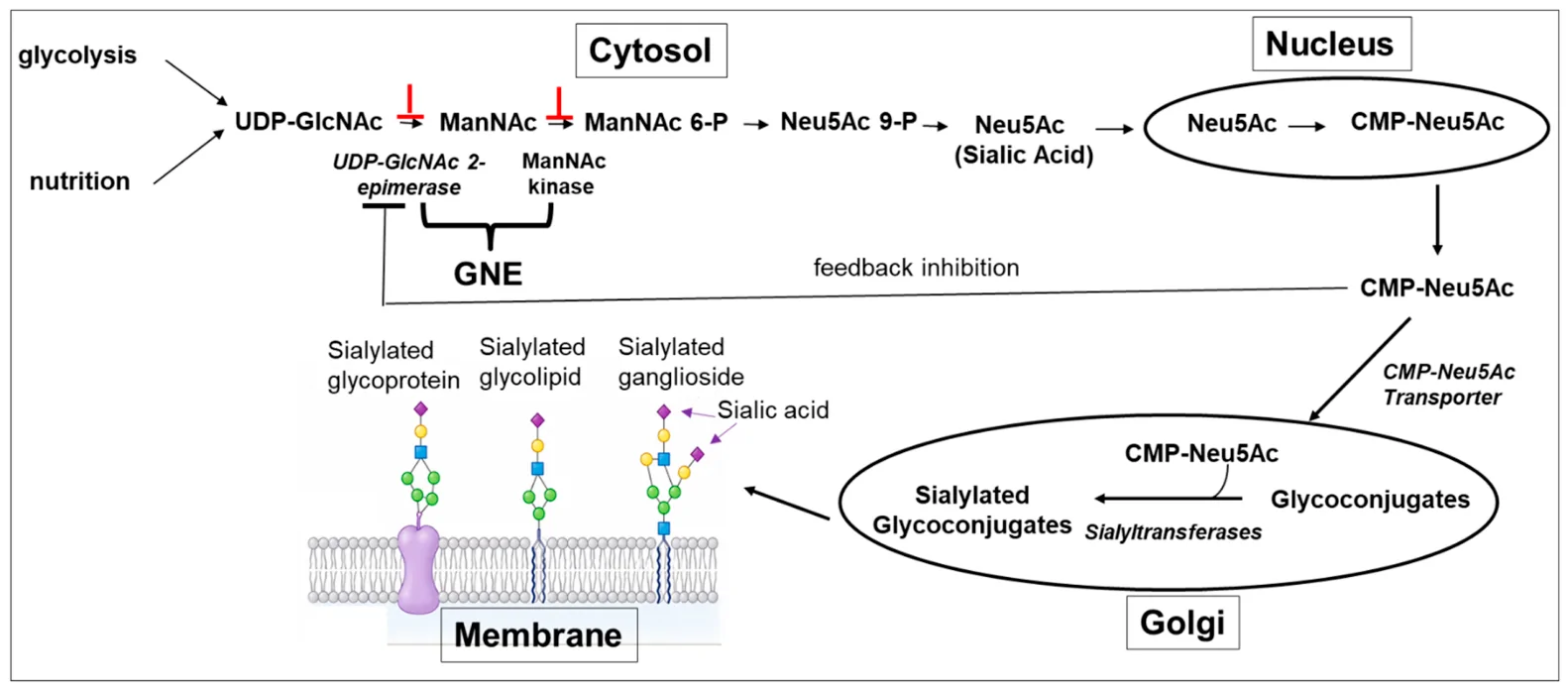
Sialic acid biosynthetic pathway in the cell. The red bars indicate the potential impaired steps resulting from pathogenic variants in GNE.

**Figure 2:**
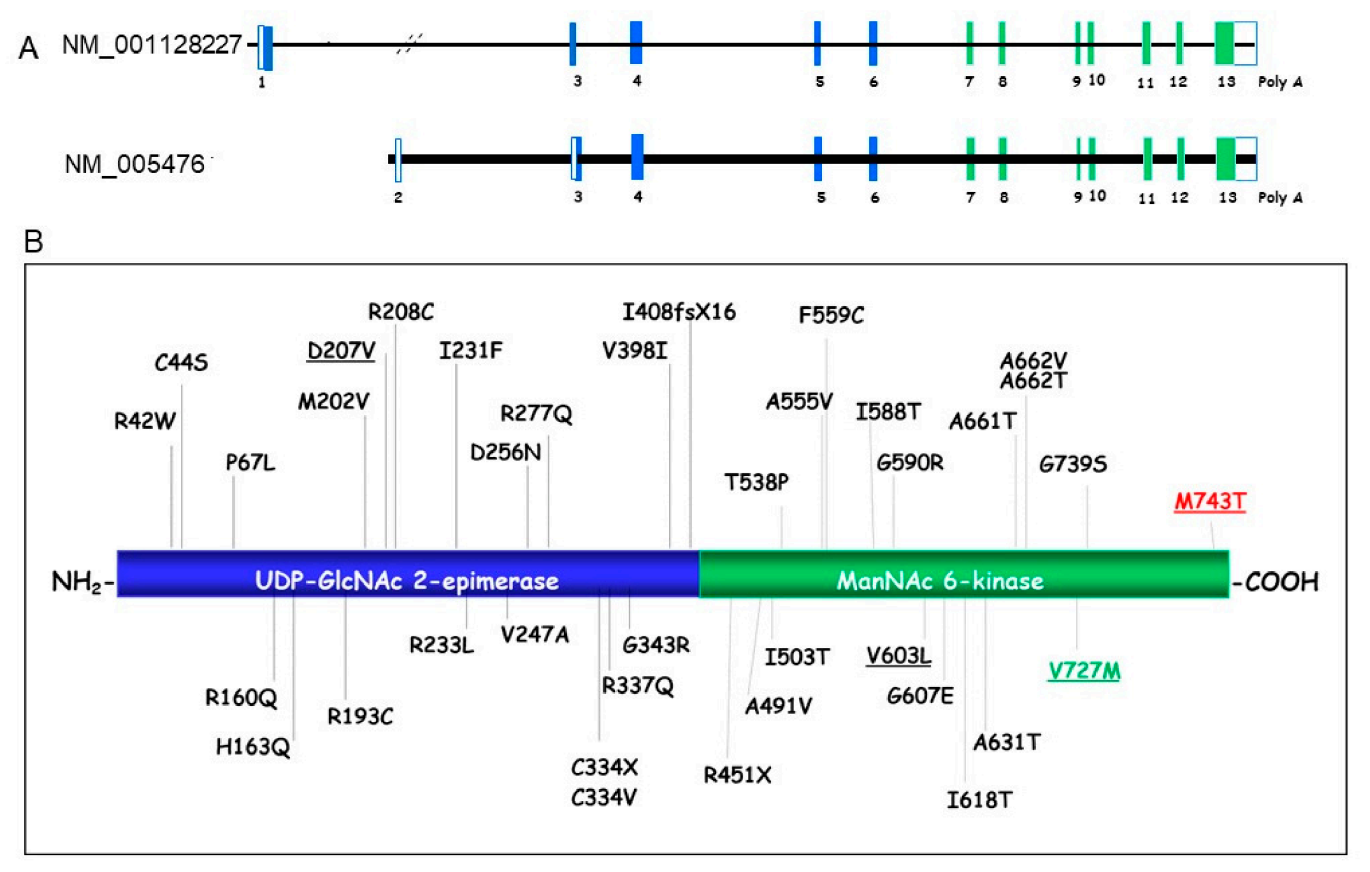
GNE gene and protein. (**A**) Schematic representation of the 2 main *GNE* transcripts. Boxes represent the exons, blue corresponds to the epimerase domain, and green to the kinase domain. Non colored spaces in exons are non-translated sequences. (**B**) Schematic representation of the GNE protein and several mutations location. Mutations mentioned in the text have been underlined (in black, founder mutations in Japan; in green, founder mutation in India; in red, Middle Eastern founder mutation).

The *GNE* gene encodes the UDP-N-acetylglucosamine 2 epimerase/N acetylmannosamine kinase protein, a bifunctional enzyme catalyzing the first two rate-limiting steps in the biosynthesis of sialic acid [15,16]. N-acetylneuraminic acid (Neu5Ac), the predominant form of sialic acid in humans, is a terminal monosaccharide critical for glycoprotein and glycolipid function [17,18] (Figure 1). GNE is ubiquitously expressed in most tissues, at relatively high levels in liver and placenta, but notably at very low levels in muscle [19]. GNE is a protein composed of 753 amino acids in its longest form. Based on the mRNA sequence, it includes 13 potential exons. Eight potential isoforms have been defined [20], all differing at their 5′ terminus and ending at the same 3′ terminus. However, only isoform 1 (722 aa) and isoform 2 (753aa), which differ by 31 amino acids at the N terminus, retain both the full epimerase and the full kinase domains (Figure 2). All the remaining potential isoforms are supposedly degraded at the RNA stage by RNA decay. Furthermore, Western blots can detect only these two isoforms, of about 79 kd, but it is difficult to distinguish between them. The full sequence of the purified protein is also difficult to achieve due to its precipitation at relatively low concentration; no crystallization of either isoform has been possible to date. De facto, most functional studies have been related to isoform 1, which up to 2014 was the only isoform known to the scientific community. From 2014 onwards, the reference sequence data shifted from transcript variant 2 (NM_005476), which (illogically) encodes the protein isoform 1, to transcript variant 1 (NM_001128227) coding for isoform 2 [21]. Notably, to date, only 2 patients have been reported with a mutation in the first “new” exon of GNE (expressed only in the longest form), a deletion affecting the 31 additional amino acids [22,23].

GNEM behaves as a classical monogenic recessive condition. The vast majority of pathogenic variants in the *GNE* gene of GNEM patients are missense point mutations, mostly compound heterozygotes, but homozygotes patients are also found, especially in inbred communities. In particular, there are several clusters of founder mutations [24], the most important one (as for the number of patients and its clinical variability) being M743T, also named as the Middle Eastern mutation (Figure 2). This has been the first pathogenic variation detected in such patients which has led to the identification of *GNE* as the gene responsible for this condition [1]. To date, more than 300 different mutations causing GNEM have been recognized. The updated various mutations’ list of *GNE*-associated myopathy can be found in online variant pathogenicity databases such as Leiden Open Variation database (LOVD) (https://www.lovd.nl) or Human Gene Mutation Database (HGMD) (https://www.hgmd.cf.ac.uk). Insertions, deletions, intronic variants and splice site mutations have also been reported [6,25]. Rarely point mutations causing a stop codon or deletions of 1 or 2 base pairs (and even larger DNA regions)—resulting in frame shift and premature termination codon—have been detected in GNEM [26,27,28]. However, these ‘nonsense’ mutations have been observed in only one allele of the affected individual, the second one being a more ‘classical’ missense mutation. To date, no cases with a double null mutation have been encountered, thus supporting the hypothesis that the complete lack of GNE is incompatible with life in humans as it is in animal models. Importantly, in few individuals with a typical GNEM clinical picture, only one pathogenic variant in one *GNE* allele has been recognized, the second one being unaccounted for (personal observations, unpublished results). This most likely reflects the limitations of the methodologies used for mutation detection. Exon sequencing can detect only changes in coding regions, however mutations located in regulatory regions of the gene have been reported in few GNEM cases [29,30,31,32], emphasizing the need for deeper or targeted sequencing techniques, such as whole genomic sequencing (WGS), copy number variants (CNV), Next Generation Sequencing (NGS), RNA sequencing or array comparative genomic hybridization (aCGH).

Although GNEM is considered a monogene recessive disorder, we are aware of 2–3 cases of individuals carrying two ‘classical’ *GNE* mutations remaining asymptomatic even at their 7th or 8th decade of life. Two of these individuals are from families with the founder M743T mutation, with siblings presenting the typical muscular disease [10,11]. A third asymptomatic subject, homozygote for the D207V mutation, has been described in a Japanese family [33]. The proposed explanation in that Japanese case has been that the D207V mutation is probably very mild pathogenically. The frequency of homozygous patients for this specific variant is much lower than expected given the frequency of this mutated allele in the Japanese population, thus raising the possibility that this particular mutation is very mild and is pathogenic only in combination with different other mutations. This could be due to the location of the variant at a site which does disrupt the oligomerization structure of the protein, without compromising its catalytic function (see Section 4). In contrast, the Middle Eastern asymptomatic cases cannot be explained according to this ‘mild mutation’ hypothesis, since all diagnosed patients in this population (>200 world-wide) carry the same homozygote M743T mutations and are clearly affected (some with a severe course) even in the very same family of the asymptomatic individuals. Our hypothesis is that protecting factors are expressed in these asymptomatic individuals which prevent the development of the myopathy. Due to the restricted number of biological resources, only one limited study attempting to unravel such factors has been reported [11]. It should be noted that the detection of these asymptomatic cases in affected families has occurred during the gene search process (through linkage disequilibrium studies) in which all family members were tested [1]. Since the discovery of the gene, only affected individuals are being specifically tested. Therefore, in spite of the information available from sequencing databases, which to date has not revealed healthy individuals carrying biallelic variants in the *GNE* gene, we should be aware of the possible occurrence of additional unrecognized asymptomatic individuals which are homozygotes, or even compound heterozygotes for some *GNE* mutations. Furthermore, studies exploring a genotype/phenotype correlation are not conclusive, most likely because of the limited number of patients available with most mutations. As mentioned, some mutations seem to be mild: in addition to the D207V Japanese mutation, the V727M Indian founder variant is most frequently found in a compound heterozygote manner, in spite of an allele frequency expected to result in several homozygote patients, thus suggesting that this mutation causes very mild symptoms or is even asymptomatic [34,35].

## 4. Biochemistry

Structurally and functionally, GNE has two well-defined enzymatic domains of roughly about the same size: the epimerase domain is defined from amino acid 1 to amino acid 378 and the kinase domain from 410 to 684 [36]. More than 300 reported pathogenic variants in GNEM span the entire *GNE* sequence, with patients carrying two mutations either in the epimerase domain, in the kinase domain, or one mutation in each domain. Both activities (epimerase and kinase) are related to the dynamic quaternary structure of this protein which has the ability to form oligomeric structures. Tetrameric/hexameric GNE has a full enzymatic activity while the GNE dimer form can work only as epimerase.

GNE is the rate-limiting enzyme in the biosynthesis pathway of sialic acid [15,37]. The first substrate of the epimerase activity of GNE in the cytoplasm is UDP-N-acetylglucosamine, which becomes N-acetyl mannosamine, subsequently phosphorylated to N-acetyl mannosamine 6 phosphate by the kinase function of GNE [36]. At this step, two more enzymatic reactions are necessary to get the final product N-acetyl neuraminic acid, also known as sialic acid (Figure 1). After entering the nucleus, NeuAc is activated by a third enzyme, and reaches the Golgi apparatus where these activated molecules (CMP-NeuAc) are incorporated as the last sugar into the glyconjugate chains (proteins and lipids) before they travel to the membrane. To note, an excess of CMP-NeuAc in the cytoplasm has a feedback effect on the epimerase function, exerted by a direct interaction between these two molecules on a specific allosteric region of the epimerase domain, between amino acids 294–297. Mutations at these specific sites of *GNE* result in the abrogation of the feedback feature and in an excess of sialic acid production, causing an extremely rare disorder known as sialuria, which manifests with mild coarse facies, slight motor delay, hepatosplenomegaly, delayed skeletal development, microcytic anemia, and mild intellectual impairment [38]. Myopathy is not part of the reported clinical picture. Interestingly, while the epimerase function of GNE is the only epimerase function known to act on UDP N acetylglucosamine (UDP-GlcNAc) in the cell, an additional kinase function, N acetyl glucosamine kinase (NAGK) which uses N acetyl glucosamine as its main substrate, can also phosphorylate N-acetylmannosamine [39]. This enzyme seems to be intact in GNEM patients [40].

Given the importance of sialylation in cellular signaling, membrane stability, and protein interactions, disruption of this pathway may have widespread cellular consequences. A defective GNE protein could have a reduced enzymatic activity that results in lower sialic acid levels and lower sialylation of membranes. This effect has been shown in humans and in animal models of the disease. However, proving the ‘obvious’ hypothesis that reduced sialic acid is the cause of muscle defective function and pathology has been difficult, and the mechanism of GNE Myopathy is still elusive (see below). Indeed, defects in several of the additional enzymes along the sialic acid pathway cause various distinct sialylation disorders, but do not result in muscle pathology [41].

## 5. Animal and Cellular Models

The results from animal studies show that GNEM models are difficult to interpret, especially in relation to the mechanism of GNEM, and difficult to use in evaluating therapies. *Gne* knock out (KO) is embryonically lethal in mice at E9-10 days [42], in line with the fact that no patient has been identified carrying two null mutations. The initial potential disease models were knock-in mice carrying either the most frequent M743T Middle Eastern mutation [43,44] or the V603L East Asian mutation [45]. These knock-in mice died a few days after birth from severe kidney disease (without a muscle phenotype at this early age). Notably, GNEM patients have no associated renal disease. A transgenic model carrying multiple copies of the D207V mutation on a *Gne* KO background [46] showed impaired muscle function with some muscle histologic changes similar to those in the human disease. The disease parameters were ameliorated by oral sialic acid or its derivatives/precursors molecules [46,47]. These findings were the main basis of the human sialic acid oral therapy trials, which were either negative or had modest effect (see Therapy Trials below). Difficulties in reproducing the transgenic/KO model in other laboratories has limited its usefulness for further research. Furthermore, its genetic characteristics are very different from the human disease genotype, preventing its use in gene therapy research [48,49].

In order to further understand GNE functions, various conditional *Gne* KO models were generated. Interestingly muscle-specific as well as muscle-and-liver-specific conditional *Gne* KO mice did not show any muscle pathology or motor dysfunction during a 2 year follow-up period [50], in spite of very low levels of sialic acid, raising the hypothesis that other supporting tissues/cells of muscle fibers contribute to muscle dysfunction in the disease rather than muscle cell abnormality. In contrast, conditional whole body *Gne* KO produced very early severe neurological phenotype (irritability, unusual postures), thrombocytopenia and short lifespan [51] with no sign of muscle pathology. In this report, only repeated massive intramuscular injection of AAVCMVCre (to obtain a systemic *Gne* KO in muscle), but not of AAVMCKCre (which result in *Gne* KO only in muscle fibers) did cause the appearance of central nuclei in the injected muscle 12 weeks after the first injection, raising again the question whether this is due indeed to *Gne* KO in the supporting cells other than muscle fibers.

Notably, a *gne* KO model in zebrafish showed survival of at least 8 days post fertilization, and several muscle defects already from day 5 [52], with compromised fiber organization. Sialic acid supplementation did not rescue the larvae phenotype and did not prolong their lifespan. RNA sequencing of these *gne* KO fish pointed also to the involvement of *gne* in cell cycle and DNA damage/repair processes. This model is of interest since it could be used for pharmacological small molecules screening.

Several in vitro cellular models have also been established. Primary muscle cells from GNEM patients’ biopsies and iPSC patients-derived cells have been cultured [53,54,55,56,57,58,59]. In addition, murine muscle C2C12 and Sol8 cell lines and even HEK (kidney derived human epithelial cells) were engineered with *GNE* mutations or *GNE* KO (interestingly *GNE* KO is not lethal in cells) and examined for different parameters, including multiple “omics” analyses, RNA sequencing and proteomics [60,61,62,63,64,65,66,67,68,69]. Although no straightforward pathway that could explain the pathomechanism of GNEM has clearly emerged from these analyses, multiple insights have come out from these studies, pointing to the involvement of GNE in several metabolic pathways. Defective myogenesis, increased oxidative stress, and mitochondrial and DNA damage/repair impairment have been recognized, as well as defects in autophagy. In addition, features related to the cell cytoskeleton were also compromised—among them the specific binding of alpha actinin to GNE [70]—and, in general, cell signaling has been shown to be broadly affected. In some of the above-mentioned cells, there have been attempts to directly/indirectly rescue the defective pathways. Two questions arise from these findings: Are these features the result of the defective sialylation profile of downstream glycoconjugates? Are they direct functions of GNE, independent of the sialylation pattern. In the latter case, supplementation with sialic acid or precursors would not correct the disease pathomechanism (Table 2).

## 6. Mechanisms

When the gene defect in GNEM was identified, the immediate thought was that this myopathy is a ‘simple’ metabolic disorder due to reduced sialic acid production via the above-described pathway (Figure 1). Reduced sialic acid production should lead to hyposialylation of most tissues, including muscle fibers (either membrane or internal cellular organelles). Indeed, several studies have shown that a defective *GNE* gene leads to reduced sialylation of muscle membrane in biopsy or cell cultures from affected patients or animal and cellular models [46,47,62,71,72]. This was the fundamental basis for the various therapy trials in GNEM (see Section 7).

There are few facts that do not support the ‘straightforward’ conclusion of a ‘simple’ enzymatic deficiency as the sole (or the main) mechanism of the muscle fiber disease in human GNEM. Although it is technically difficult to measure directly sialic acid in muscle fiber, various lectin stains that evaluate membrane sialylation in GNEM muscle cells showed reduced but not absent sialylation [71,72]. However, investigations of the GNE enzymatic functions showed that the extent of the activity reduction in lymphocytes, myoblasts and myotubes of GNEM patients varied only between 30% and 60% [73,74], making it difficult to explain how such partial enzymatic activity reduction could lead to markedly reduced sialic acid production. Marked GNE deficiency has not been observed in GNEM patients, in fact the GNE protein is expressed at equal levels in patients and normal control subjects [75]. In general, metabolic myopathies due to recessive gene defects show phenotypes when levels are lower than 5–20% and unaffected carriers would have levels of 50%. Attempts to detect a potential specific glycoconjugate in which the GNE-related reduced sialylation would be more critical were unsuccessful [76,77,78].

Our reluctance to fully adopt the sialic acid deficiency hypothesis in GNEM also comes from difficulties in interpreting the results of several animal and cellular models with *Gne* defects. In mice *Gne* KO leads to intrauterine death probably due mainly to brain hemorrhages [79], making it impossible to study muscle tissue. Transgenic animals with multiple copies of defective *Gne* yielded a late onset myopathy with muscle histopathology similar to the one observed in human disease [46]. In this model relative rescue as a response to various sialic acid precursors supported the hyposialylation mechanism. However, there is no genotypic similarity between this transgenic mouse model and the human disease. As mentioned above, knock-in mice models [43,44,45] with homozygous mutations mimicking the human genotype, showed an early kidney disease but no muscle phenotype pathology. Recently, a postnatal conditional mouse model with induced *Gne* knock out in muscle, showed clear muscle hyposialylation but no muscle disease [50]. The above experimental observations support the controversy about the role of sialic acid deficiency in the pathogenesis of GNEM. The disappointing results of the human trials (see Section 7) are also a reason to consider another pathomechanism for GNEM.

A hypothetical theory could be that the defective (mutated) GNE (either mRNA or protein) exerts a toxic effect on muscle cells. According to such theory the muscle fiber hyposialylation would be an epiphenomenon. One observation to support this theory is the fact that dramatic hyposialylation of muscle in mice does not result in muscle pathology [50] and, further, that only a ‘large load’ of defective *Gne* as in the transgenic mouse model [46] leads to myopathy. This hypothesis remains to be proven, but a recent cell study examined a double genetic therapy protocol suppressing the mutated GNE and providing a wild-type normal gene [80], a process that is in agreement with a ‘toxic’ effect of the defective GNE.

As mentioned above, several biochemical changes reported mostly in GNE-deficient cells could point to additional primary or secondary mechanisms that may contribute to muscle pathology.

## 7. Therapy Trials (Past and Future)

According to the prevailing theory, GNE enzyme activity is defective (but not absent) in patients, leading to a reduced production of the product, sialic acid, and to subsequent hyposialylation in muscle cells that would cause muscle cell degeneration. Providing muscle tissue with high amounts of sialic acid or its intermediates in the sialic acid synthesis pathway would lead to increased muscle sialylation and prevention or correction of muscle weakness in GNEM.

Five important human therapy trials in GNEM were based on the assumption that increased oral supplementation of sialic acid or its metabolic precursor could have a positive effect in improving or slowing the progression of GNEM (Table 3). Three trials used oral extended-release sialic acid (SA-ER). A preliminary phase 2 [81] suggested a positive effect as measured by an upper extremity composite (UEC) score of muscle force and a GNEM patient-reported outcome (GNEM-FAS), both developed specifically for clinical trial outcome measures [82]. This study was important because it defined an outcome measure that was used as a primary goal in following studies, but was not the definite phase 3 of this group and thus is not mentioned in Table 3. A larger controlled phase 3 trial using the same SA-ER product and UEC as primary outcome did not show a positive effect [83]. A later trial in Japan using a very similar supplementation product (SA-ER) showed slowing of UEC score deterioration after 48 weeks [84] and a follow-up smaller study of 72 weeks seemed to confirm the observation [85]. These two Japanese studies led to an approval of this compound as drug therapy for GNEM in Japan. A small preliminary pilot trial of 6′-sialyllactose (6′-SL), a metabolic substance that enables sialic acid production outside the regular metabolic pathway, had a positive signal in phase 1/2 study in South Korea, as measured by grip force deterioration [86]. This small study identified MRI measurement of fat fraction in the posterior hip muscles and lectin staining of blood cells as potential markers of positive effects of this compound. Another major trial used the second intermediate compound in the normal synthetic pathway of sialic acid, N-acetyl mannosamine (ManNAc). This was a large scale phase 3 trial over 2–3 years. This trial used a different primary outcome measure of muscle power (quantitative muscle assessment, QMA, for 5 selected muscles). Unfortunately, it was recently reported as negative [87].

Why were these metabolic supplement studies negative or showing only mild/modest effect? There are several possibilities besides the obvious one that lack of sialic acid by itself is not the main cause of weakness in GNEM:

1. The penetration of oral supplementation drugs is not efficient enough in human muscle fibers, unlike the findings in mouse models of GNEM. Increased sialylation of muscle tissue in these human trials was not demonstrated. Relying on doses equivalent to the ones used in the mice studies would mean much higher doses in human trials that could be either intolerable or toxic in humans.
2. There could have been potential problems with the trial’s design:
  a. The trials were too short. GNEM is a chronic, slowly progressive disease, and a one-year (48-week) therapy trial is not sufficient to demonstrate a measurable effect. However, the ManNAC trial was two years long (with an extension to 3 years) and longer studies are difficult to maintain financially.
  b. A mix of patients with various mutations and different rates of disease progression was included, potentially masking positive effects. In general, the mutation nature has been postulated to be responsible for only about 20% of the disease severity [88], but this mixed patient population may still add an additional ‘masking’ effect. Also, as mentioned above, several studies [33,34,89] suggested that at least two frequent pathogenic variants in Asia—Japan, China and India—(D207V and V727M) could be benign, and potentially there could be others. In these trials a pretrial selection was not (or could not) be balanced in the patients’ recruitment (placebo control vs. active therapy) because GNEM is a very rare disorder.
  c. Another problem with the selection of the patients for the trials was the stage of the disease. It seemed that patients with a better score (>200 m) on the six-minute walk test (6MWT) could show a better effect. This would suggest that the effect of oral supplementation could be useful only if given at the early stages of the disease. The ManNAc trial also used an exclusion criterion of severely advanced cases. Notably, in our cohort of more than 150 patients carrying the same ‘Middle Eastern’ homozygote mutation (M743T) some have a rather mild course, maintaining ambulation of flat ground into the 6–7th decade of life while others become severely paralyzed from the neck down in less than 10 years. Age at onset is possibly a marker for further course severity and it is suggested to use it as a ‘balancing’ factor in the assignment of the treated vs. placebo group.
  d. The endpoints of the trials were not sensitive enough to show significant changes. The primary endpoint in the initial studies was an upper extremity composite score (UECS), a calculated sum of upper limb power measured by hand-held dynamometer (HHD). These power measurements should be performed by a very well-trained examiner (usually a physiotherapist), and multicenter large trials are not homogeneous by nature. The UECS was the primary outcome measure in the large negative international study [83] but was also used in the positive Japanese study [84], so this test may not be an ideal outcome measure.

Other outcome measures have their own issues with suitability to GNEM. The ‘traditional’ six minute walk test in a disorder with unusual muscle weakness distribution where patients’ gait on flat ground is typically better preserved compared with their other legs’ functions seems less suitable compared with other limb girdle myopathy types. The information on MRI changes during the course of GNEM is limited. Previous semi quantitative observations are only available in the trial performed in Korea [86]. The observations were made at two levels (midthigh and midcalf) but the exact definition of the location is not clear. There is currently no data about the relationship between MRI and function in GNEM. A patient-reported outcome was developed specifically for GNEM (GNEM-FAS); however, there was low correlation with the upper extremity composite score (UECS) which was the major endpoint in following studies [85]. The ManNAC trial (named MAGiNE) used a complicated machine assessment of muscle power for each muscle. The study design was not statistically planned for showing an effect on the rate of disease progression but more on improvement of force. A trial aimed at assessing course of disease would theoretically need a much larger group of patients with a longer time-course. The ManNAc trial seemed to calculate deterioration of each muscle overtime so apparently this was not the cause of drug failure in that trial.

There has been only one single-patient compassionate trial of genetic therapy in GNEM. The patient was given intravenous injections of incremental doses of wild-type *GNE* over a period of 13 months [90]. The DNA vector was coupled to a CMV promoter and delivered systemically in a liposomal package (Lipoplex). The effect on muscle function was minimal but the patient was in an advanced phase of the disease and much strength recovery could not be expected. However, 72 h after the highest dose, expression of wild-type GNE and increased sialylation in muscle was demonstrated by biopsy. This single-patient trial showed a possible proof-of-principle for *GNE* delivery method, although it is expected that infusions will have to be intermittently repeated using this delivery mode. Genetic therapy seems to us to be the next step in therapy trials but the methods are still at the preclinical stages.

The above-discussed unresolved issues in the understanding of GNEM pathomechanisms, together with the experience gained from previous clinical trials, lead to several potential avenues for future therapeutic development:

1. Improved sialic acid supplementation approach. If one of the reasons for lack of major effect in this metabolic approach is the limited penetration of the various derivatives into the muscle fiber, improved pharmaceutics can be designed. This approach led Ultragenix (the company that supported the first SA-ER studies) to recently announce a trial of modified sialic acid (SA) prodrug: “prodrug composed of SA and a hydrophobic fatty acid tail that enhances efficient delivery to muscle as compared to naturally occurring SA. Based on preclinical data, the fatty acid tail improves distribution to muscle and other tissues and supports more efficient uptake and release of SA within muscle”. Their approach received FDA approval for a Phase 1/2 trial, and it remains to be seen whether such improved metabolic efficiency will result in a better clinical effect (NCT07511556). A different metabolic approach was tried by above-mentioned Korean research team that used a biochemical source of sialic acid outside the common synthesis pathway affected in GNEM- 6′-sialyllactose (6′-SL) [86].

The currently observed negative or modest effects of sialic acid therapy trials in GNEM is a ‘warning’ sign that ‘simple’ oral supplementation may not suffice.

2. Genetic therapy. The ‘simplest’ approach would be to supply the muscle cells of GNEM with wild-type *GNE* using AAV as the vector (a platform used in other neuromuscular disorders trials). Research in mice (wild-type as well as genetically modified) has shown that this mode of delivery is efficient in supplying muscle and other cells with GNE mRNA [49,91], but, unfortunately, the lack of a reliable animal model hampers the attempts to reach clinical trial usage. More sophisticated methods will be adding to this WT *GNE* delivery an additional muscle growth factor, for example, follistatin (Paul Martin, Genosera, personal communication) to prevent muscle atrophy and stimulate muscle growth.

In the theoretical case that the mutated *GNE* by itself is toxic to the cell (discussed above), another ‘double genetic therapy’ approach could be considered: delivery of wt *GNE* associated with mRNA inhibition of the mutated gene in the affected patients [80]. This approach as well as CRISPR methods to correct specific mutations of *GNE* will have to be tailored to each mutation, but could be used in populations of GNEM patients with founder mutations.

In all these approaches, the main problem remains the delivery efficiency, resulting in potentially toxic high viral doses: there are efforts to develop AAV envelope and promoter systems targeting muscle more specifically [92,93], thus potentially allowing a reduction in the viral vector dosages. Also, the usage of liposome-based platforms is being explored [94].

3. Additional drugs. Using drugs that affect possible downstream effects of impaired GNE (either due to lack of sialic acid or to lack of an additional unknown GNE function). As mentioned in the Section 6, these could be inhibitors of apoptosis or of toxic ROS, among others. Even if such therapies do not deal directly with the primary defect, they could address and alleviate possible secondary effects of the disease.

## 8. Conclusions

Twenty-five years after identifying the genetic basis of GNEM, there are still several gaps in understanding the pathomechanism of this myopathy that complicate further therapy development. The main controversy in our view is whether GNEM is indeed due to muscle hyposialylation. We are also still facing a few enigmas: Why do the quadriceps remain so little involved even in the very late stages of the disease? What is the mechanism that protects the asymptomatic homozygous patients with typical *GNE* mutation? And how does the very rare *GNE*-related thrombocytopenia correlate with GNEM?

The identification of pathogenic variants in the GNE gene has significantly advanced the diagnosis and genetic counseling of GNE Myopathy (GNEM). Furthermore, it has expanded our understanding of the role of sialic acid in skeletal muscle function and disease pathogenesis. A major focus of future research will be the development of effective gene-based therapies capable of addressing the diverse pathogenic mechanisms proposed in this review.

## Figures and Tables

**Table 1:** Clinical features of GNE Myopathy.

Common Manifestation	Unusual Features	Rare Features
Young adult onset, mean around 30 years of age First sign: drop foot (usually bilateral, can be asymmetric) Progression to involve proximal leg musculature (mainly hip flexion) Quadriceps sparing even at advanced age Weakness of paraspinal muscles Prolonged preservation of ambulation on flat ground No shortening of life expectancy Mild elevation of serum CK (<X5 of normal values) Myopathy with spontaneous activity in calf muscles on EMG	No quadriceps sparing (<5%) Onset in proximal lower limb muscles Onset in hands Onset in early teens or 5th decade Mild facial weakness Abdominal wall weakness	Cardiomyopathy (after r/o all other causes) Reduced respiratory functions (usually in wheelchair bound) Macrothrombocytopenia

**Table 2:** Animal and cellular models for GNE Myopathy.

	GNE Genotype Variation	Phenotype	Comments	Reference
**MICE**	GneKO	embryonically lethal		Schwarzkopf et al. 2002 [42]
	GneM743T	early death from severe kidney impairment	generated by homologous recombination	Galeano et al., 2007 [43]
	GneM743T	variable phenotype from early death to normal lifespan	generated by homologous recombination	Sela et al. 2013 [44]
	GneV603L	early death from severe kidney impairment	generated by homologous recombination	Ito et al., 2012 [45]
	TgD207V on GneKO background	recapitulates several characteristics of GNE Myopathy	phenotype diluted with generations	Malicdan et al., 2007 [46]
	Conditional muscle GneKO	no phenotype		Harazi et al., 2024 [50]
	Conditional muscle &liver GneKO	no phenotype		Harazi et al., 2024 [50]
	Conditional whole body GneKO	early death neurological impairment severe thrombocytopenia		Lam et al., 2025 [51]
**ZEBRAFISH**	tg M743T	no phenotype		Livne et al., 2022 [52]
	gne KO	death at 8–10 days post fertilization; decreased movement and heart rate; compromised muscle fiber organization; DNA damage/repair pathway defects		Livne et al., 2022 [52]
**CELL CULTURES**	Patients’ myoblasts with	differentiation defects, impaired apoptosis	homozygotes and compound	Amsili et al., 2007 [53]
	Diverse missense mutations	cytoskeletal structure, oxidative phosphorylation; autophagy	heterozygotes	Harazi et al. 2015, Eisenberg et al. 2008, Sela et al., 2011, Cho et al., 2017 [54,55,56,57]
	Patient’ s iPSCs	autophagy defects	compound heterozygote	Schmitt et al., 2022 [58]
	Human PSCs	autophagy defects	base editing	Kim et al., 2026 [59]
	Murine Sol8 GneKO	cell cycle defects; impaired differentiation; DNA damage/repair defects; sarcomere organization		Ilouz et al., 2022 [60]
	Murine C2C12 GneKO	myogenesis		Neu et al., 2024; 2026 [61,62]
	Human HEK GNE +/−	impaired signaling of various pathways including apoptosis, ROS, autophagy, ER stress	heterozygous Gne mutations	Singh et al., 2016; 2018, Yadav et al., 2022, Oswalia et al., 2024 [64,65,66,68]

**Table 3:** Major human sialylation trials for GNE Myopathy.

Drug; Dose; Time	Trial Id; Report Date	Phase	Inclusion	Number (Randomization)	Major Outcome	Result	Comments
SA-ER 6 gr/d 48 weeks	NCT02377921 2021	3	>200 m on 6MWT	89 (1:1)	UEC score	No significant improvement	International study
SA-ER 6 gr/d 48 weeks	NCT04671472 2023	3	>200 m on 6MWT	20 (1:4)	UEC score	Significant improvement	Multicenter study in Japan. Drug approved in Japan
6′SL 6 gr/d 48 weeks	No NCT 2025	Local Pilot (phase 2?)	Ambulant, <10 year duration	11 (5 drug-6 placebo)	None predefined	Less grip decline, Less fat on hip MRI	Only in Korea
ManNAc 12 gr/d 48 weeks	NCT02346461 2026	3	10–65% of predicted strength in 5 muscle groups	54 (2:1)	MVIC by QMA	No significant benefit on muscle strength decline	Reported recently in abstract form only

6MWT = 6 min walk test; 6′SL = 6 sialyllactose; ManNAC = N-acetylmannosamine; MVIC = maximum voluntary muscle contraction; QMA = quantitative muscle assessment; SA-ER = sialic acid extended release; UEC = upper extremity composite.

## Data Availability

No new data were created or analyzed in this study. Data sharing is not applicable to this article.

## Abbreviations

The following abbreviations are used in this manuscript:

GNEM	the disease;
GNE	the protein;
*GNE in humans, Gne in mice, gne in Zebrafish*	the gene.
